# Balance Performance in Irradiated Survivors of Nasopharyngeal Cancer with and without Tai Chi Qigong Training

**DOI:** 10.1155/2014/719437

**Published:** 2014-09-11

**Authors:** Shirley S. M. Fong, Louisa M. Y. Chung, William W. N. Tsang, Joyce C. Y. Leung, Caroline Y. C. Charm, W. S. Luk, Lina P. Y. Chow, Shamay S. M. Ng

**Affiliations:** ^1^Institute of Human Performance, The University of Hong Kong, Pokfulam, Hong Kong; ^2^Department of Health and Physical Education, The Hong Kong Institute of Education, Hong Kong; ^3^Department of Rehabilitation Sciences, The Hong Kong Polytechnic University, Hong Kong; ^4^Division of Nursing and Health Studies, Open University of Hong Kong, Hong Kong; ^5^The Association of Licentiates of the Medical Council of Hong Kong, Hong Kong

## Abstract

This cross-sectional exploratory study aimed to compare the one-leg-stance time and the six-minute walk distance among TC Qigong-trained NPC survivors, untrained NPC survivors, and healthy individuals. Twenty-five survivors of NPC with TC Qigong experience, 27 survivors of NPC without TC Qigong experience, and 68 healthy individuals formed the NPC-TC Qigong group, NPC-control group, and healthy-control group, respectively. The one-leg-stance (OLS) timed test was conducted to assess the single-leg standing balance performance of the participants in four conditions: (1) standing on a stable surface with eyes open, (2) standing on a compliant surface with eyes open, (3) standing on a stable surface with eyes closed, and (4) standing on a compliant surface with eyes closed. The six-minute walk test (6MWT) was used to determine the functional balance performance of the participants. Results showed that the NPC-control group had a shorter OLS time in all of the visual and supporting surface conditions than the healthy control group *(P* < 0.05). The OLS time of the TC Qigong-NPC group was comparable to that of the healthy control group in the somatosensory-challenging condition (condition 3) *(P* = 0.168) only. Additionally, there was no significant difference in the 6MWT distance among the three groups *(P* > 0.05). TC Qigong may be a rehabilitation exercise that improves somatosensory function and OLS balance performance among survivors of NPC.

## 1. Introduction

Nasopharyngeal cancer (NPC) is a rare malignancy in North America and Europe (incidence rate: 1 per 100,000) but is common in endemic areas that include the southern part of China, Southeast Asia, and North Africa. The incidence rate ranges from 25 to 50 per 100,000 in these endemic regions [1]. In terms of the medical management of NPC, radiotherapy has so far been the mainstay of treatment [2]. Although it is generally believed that the inner ear is not affected by radiation at the dosage commonly used for therapy [3], evidence suggests that radiation-induced inner ear damage does occur in animals [4] and human beings treated for NPC [5]. As the vestibular apparatus located in the inner ear is responsible for body balance [6], it is possible that survivors of NPC after radiotherapy have vestibular dysfunction and postural control deficits. To date, only one study has examined the standing balance performance of irradiated NPC patients. Using posturography and a standard Romberg position, postural sway in bipedal stance was quantified, and the results revealed that postural control was preserved in patients with NPC [5]. However, the authors adopted a double-leg standing posture, which may not have been challenging enough for the participants because of the large base of support [7]. Postural control and sensory deficits might be manifested when NPC survivors stood on one leg (with a smaller base of support). Indeed, chemotherapy could also affect functional balance performance and sensory organization (i.e., visual, somatosensory, and vestibular senses) in postural control [8]. Therefore, our first hypothesis was that the one-leg-stance (OLS) balance in sensorially challenging conditions (achieved by changing the visual input or support surface stability) and the functional balance performance of NPC survivors would be inferior to those of healthy controls.

Tai Chi (TC) Qigong, which integrates the essence of TC and Qigong, is a Chinese physiotherapeutic approach that consists of breathing exercises coordinated with slow body movements and balance training in an upright posture. It is a type of mind-body exercise and is particularly suitable for patient populations because it is relatively simpler and more repetitive than the traditional TC forms [9, 10]. According to a recent comprehensive review, a growing body of evidence suggests that these mind-body exercises, TC/Qigong/TC Qigong, can improve body balance (e.g., OLS) in patient populations [11]. Therefore, our second hypothesis was that NPC survivors undergoing TC Qigong training would have better OLS balance performance in different sensory conditions than, and superior functional balance performance to, their nontrained counterparts. Their balance ability might even reach the level of healthy individuals. Based on these hypotheses, the main purpose of this cross-sectional study was to compare (1) the one-leg-stance time in different visual and support surface conditions and (2) the six-minute walk distance among TC Qigong-trained NPC survivors, untrained NPC survivors, and healthy individuals.

## 2. Methods

### 2.1. Participants

This is a cross-sectional study. One hundred and twenty senior adults participated in the study voluntarily. Survivors of NPC with TC Qigong experience (*n* = 25) were recruited from a Qigong Association that provides TC and Qigong training for cancer survivors. Survivors of NPC without TC Qigong experience (*n* = 27) were recruited from a medical clinic and a cancer self-help group. The inclusion criteria were the following: (1) having a history of NPC (i.e., positive Epstein-Barr virus-DNA and biopsy test results) but being cancer-free during the study period; (2) having finished all cancer treatments in hospital (radiotherapy with or without chemotherapy); (3) being medically stable; (4) being between 40 and 85 years old; (5) being Hong Kong Cantonese; (6) having normal cognitive function and being able to follow instructions; and (7) having been trained in the 18 forms of Tai Chi Internal Qigong [10] for at least six months continuously (for the NPC-TC Qigong group participants only). The exclusion criteria were the following: (1) receiving alternative and complementary therapies such as acupuncture; (2) significant medical conditions such as diabetes mellitus; (3) known sensorimotor, neurological, musculoskeletal, cardiopulmonary, or vascular disorders limiting locomotion or balance performance; (4) walking with assistive device or being wheelchair-dependent; and (5) being engaged in regular exercise such as morning TC.

Sixty-eight healthy senior adults were recruited from two local community centers. They followed the same inclusion and exclusion criteria mentioned above except that they did not have any history of NPC and TC Qigong experience. Written informed consent was obtained from each participant before the data collection. All of the procedures were conducted according to the Declaration of Helsinki, and all of the experimental work was carried out with the approval of the ethics review committee of the administering institution.

### 2.2. Outcome Measures

Demographic characteristics and medical history were first obtained by interviewing the participants (Table 1). Each participant then underwent the following evaluations of balance and functional outcomes in sequence. A one-minute break was allowed between the tests to avoid fatigue.

#### 2.2.1. One-Leg-Stance Time

The one-leg-stance timed test was conducted to assess standing balance. The participants were instructed to stand barefoot on their dominant leg (1) on a stable surface (ground) with eyes open; (2) on a compliant surface (Stability Trainer, The Hygienic Corporation, Ohio, USA) with eyes open; (3) on a stable surface (ground) with eyes closed; and (4) on a compliant surface (Stability Trainer) with eyes closed. Their arms were at rest on either side of the trunk. In the two eyes-open trials, the participants were instructed to focus on a spot on a nearby wall in front of them. Close guarding was provided to prevent falls during the trials. A stopwatch was used to record the duration of standing (in seconds). The OLS time commenced when the nondominant foot left the ground and ended if the same foot touched the ground or rested against the other leg or the participants hopped on the weight-bearing leg or shifted on the weight-bearing foot or opened their eyes in the eyes-closed trials or when a 60-second OLS duration was reached [12]. A maximum of two trials was allowed for each testing condition for the participants to achieve the 60-second goal. If the participants reached the goal of 60 seconds in the first trial, no further trial was conducted for that particular testing condition. The time of the better trial of each testing condition was recorded. A longer duration indicated better balance ability [12].

#### 2.2.2. Six-Minute Walk Distance

The six-minute walk test (6MWT) was used to determine the functional balance performance of the participants [13]. In accordance with the American Thoracic Society guidelines [14], the participants were instructed to cover as much distance as possible at a self-paced walking velocity in a 30-meter unobstructed walkway within six minutes. They were allowed to rest during the test if necessary but were instructed to resume walking as soon as possible. The total distance walked, to the nearest meter, in a single trial was documented. A longer distance covered indicated a higher level of functional mobility, better balance performance, and better cardiovascular fitness [13, 15]. This test has been found to have good test-retest reliability when used in older adults (0.88 < *r* < 0.94) [16].

### 2.3. Statistical Analysis

The Statistical Package for the Social Sciences (SPSS) version 20.0 software was used to perform the statistical analyses. All of the demographic and outcome variables were presented using descriptive statistics. The normality of the continuous data was checked using the Kolmogorov-Smirnov test. One-way analysis of variance (ANOVA) was used to compare the age, weight, height, and body mass index of the three groups. A Chi-squared test was used to compare the sex ratio of the groups. In addition, an independent *t*-test was used to compare the post-NPC duration of the two NPC groups. If significant between-group differences were found in any of the demographic variables, these outstanding outcomes were treated as covariates in the subsequent balance and functional outcome analyses.

For the analyses of the balance and functional outcomes, one-way analysis of covariance (ANCOVA) was used to compare the differences among the three groups. Bonferroni tests were used to analyze the data post hoc as necessary. A significance level of 0.05 (two-tailed) was set for all of the statistical tests.

## 3. Results

### 3.1. Demographic Characteristics

The characteristics of the three groups of participants are presented in Table 1. As there were significant between-group differences (*P* < 0.05) in weight and body mass index (BMI), these demographic variables were entered as covariates in the univariate analysis. The sex ratio was also entered as a covariate because it was marginally significant (*P* = 0.057) and, clinically, gender differences can affect balance performance [17, 18].

### 3.2. Condition 1: One-Leg-Stance on Stable Surface with Eyes Open

The results revealed a significant difference in OLS time among the three groups (*F*(2,117) = 11.912, *P* < 0.001). Post hoc analysis found that both the NPC-TC Qigong (*P* = 0.013) and the NPC control (*P* = 0.003) groups had a shorter OLS time than the healthy control group. However, no significant difference in OLS time was found between the two NPC groups (*P* = 1.000) (Table 2).

### 3.3. Condition 2: One-Leg-Stance on a Compliant Surface with Eyes Open

An overall significant difference among the three groups was found (*F*(2,117) = 11.505, *P* < 0.001). The healthy control group stood longer on one leg than the NPC-TC Qigong (*P* = 0.023) and the NPC control (*P* = 0.004) groups. The OLS time of the two NPC groups was similar (*P* = 1.000) (Table 2).

### 3.4. Condition 3: One-Leg-Stance on a Stable Surface with Eyes Closed

The ANCOVA result was significant (*F*(2,117) = 6.854, *P* = 0.002). The NPC control group stood for a shorter duration than the healthy control group (*P* = 0.007) when the eyes were closed. However, the OLS time was similar between the NPC-TC Qigong and the healthy control groups (*P* = 0.168) and between the NPC-TC Qigong and the NPC control groups (*P* = 1.000) (Table 2).

### 3.5. Condition 4: One-Leg-Stance on a Compliant Surface with Eyes Closed

The ANCOVA result was also significant (*F*(2,117) = 11.147, *P* < 0.001) in this most challenging condition. The healthy control group stood for longer on one leg than the NPC-TC Qigong (*P* = 0.018) and the NPC control (*P* = 0.001) groups. No significant difference in OLS time was found between the two NPC groups (*P* = 1.000) (Table 2).

### 3.6. Six-Minute Walk Test

There was no significant difference among the three groups in the distance covered in the 6MWT (*F*(2,117) = 1.279, *P* = 0.282) (Table 2). Therefore, post hoc analysis was not undertaken.

## 4. Discussion

This is the first study to show that participating survivors of NPC had inferior OLS balance performances in all of the visual and supporting surface conditions compared with age-matched healthy counterparts. Given that postural control requires the ability to utilize sensory inputs (i.e., somatosensory, visual, and vestibular inputs) and to generate coordinated motor outputs [19], our results suggest that survivors of NPC might have multiple underlying sensorimotor impairments in OLS balance control. Indeed, a previous study showed that breast cancer patients treated with chemotherapy demonstrated increased postural instability in bipedal stance compared with healthy controls [8]. Chemotherapy-related peripheral nervous system disorders (e.g., sensorimotor neuropathy, somatosensory, visual, and vestibular deficits) may be the contributing factors [8, 20]. In addition, irradiation of the temporal bone during NPC treatment may result in vestibular damage such as canal paresis [5] that may also affect balance performance. All of these sensorimotor impairments (side effects) resulting from radiotherapy or chemotherapy might explain the overall inferior OLS balance performance among survivors of NPC.

With TC Qigong training, the participating survivors of NPC had a similar OLS balance performance in condition 3 (i.e., stood on a stable surface with eyes closed) to that of the healthy individuals, although they were still no better than the NPC controls. As somatosensory input is the dominant sensory input for balance under a stable support surface and eyes closed condition [19], our results suggest that TC Qigong-trained NPC survivors might rely more on their somatosensory input to balance. This reliance might be because the TC Qigong-trained participants had better knee and ankle joint proprioception that might have resulted in better balance control in the OLS [21].

Ample evidence supports that TC and Qigong training can improve the use of vestibular input to balance [22–24]. For example, using the sensory organization test, Tsang et al. [23] reported that long-term TC practice may improve balance control in the elderly when there was increased reliance on the vestibular system during stance. However, in our study, because irradiation-related damage to the vestibular apparatus is irreversible [5], exercise (TC Qigong) training might not be able to remediate the vestibular deficit in survivors of NPC. Therefore, our NPC-TC Qigong group performed similarly to the NPC control group when relying primarily on their vestibular input to balance (i.e., condition 4—stood on a compliant surface with eyes closed). The nonirradiated healthy controls performed much better than the two NPC groups in this vestibularly challenging condition.

When relying primarily on visual input to balance (i.e., condition 2—stood on a compliant surface with eyes open), the healthy control group also outperformed the two NPC groups, while the two NPC groups performed similarly. This suggests that TC Qigong training might not be able to improve the use of visual input for postural control in survivors of NPC, perhaps because the vestibuloocular reflex is also disrupted in survivors of NPC who undergo radiotherapy [5], and exercise (TC Qigong training) might not be able to heal it.

Interestingly, we found that when the three sensory inputs were present and accurate (condition 1), both the NPC-TC Qigong and the NPC control groups had a shorter OLS duration than the healthy control group. In addition, both groups of NPC survivors balanced similarly in the OLS. The TC Qigong-trained NPC survivors might have been unable to match the healthy controls in terms of OLS performance because, typically, dysfunction in any one of the three senses that contribute to balance can be compensated by inputs from the other two sensory systems. For example, dysfunction of the vestibular system may be compensated by enhancing somatosensory awareness and visual attention to better balance [19]. However, two senses (vestibular and visual) out of the three might have been disrupted in our TC Qigong-trained NPC survivors due to the side effects of radiotherapy or chemotherapy, as explained. Therefore, the compensation mechanism might not have worked in this case. The NPC survivors still had an inferior OLS balance performance when they stood on a stable surface with eyes open relative to the healthy controls, even though they trained in TC Qigong regularly. Certainly, further studies should examine the functions of the individual sensory systems to substantiate this hypothesis.

The sensorimotor deficits of postural control among the survivors of NPC, both with and without TC Qigong training, were not reflected in the six-minute walk test result. This result might be because this test is too general as it assesses both the balance ability and the functional aerobic capacity of the participants [15]. Walking distance might, therefore, have been limited by the aerobic capacity rather than by the balance ability of the participants. Further studies should consider using other dynamic balance measurements, such as the Berg Balance Scale, to quantify the functional balance ability of survivors of NPC [25].

Some limitations to this study warrant comment. First, the use of a convenience sample may have introduced a self-selection bias that may threaten the internal validity of the study. In addition, our rather homogenous subject group may limit the generalizability of results [26]. Second, the study was cross-sectional in nature and thus a cause-and-effect relationship between TC Qigong training and balance performance could not be established. Third, both of the NPC groups had a rather small sample size, which may partially explain some of the insignificant findings. Finally, the OLS clinical test was used to assess balance performance. Future studies could consider using more comprehensive, objective, and accurate laboratory measures (e.g., computerized dynamic posturography) [19] to evaluate the balance ability thoroughly of survivors of NPC.

## 5. Conclusions

Irradiated survivors of NPC had inferior single-leg standing balance performance relatively to the healthy individuals. The survivors of NPC who were trained in TC Qigong might have relied more on their somatosensory input to maintain single-leg standing balance. Their one-leg-stance time on a stable surface with eyes closed was comparable to that of healthy individuals. However, survivors of NPC, both with and without TC Qigong training, had a shorter OLS time than healthy controls when they stood (1) on a stable surface with eyes open, (2) on a compliant surface with eyes open, and (3) on a compliant surface with eyes closed. The six-minute walk distance was comparable among NPC survivors with and without TC Qigong training and healthy controls. Our results hint that TC Qigong might be a potential rehabilitation exercise to improve the somatosensory function and single-leg standing balance performance of survivors of NPC.

## Figures and Tables

**Table 1:** Characteristics of the participants.

	NPC-TC Qigong group (*n* = 25)	NPC-control group (*n* = 27)	Healthy-control group (*n* = 68)	*P*
Age (year)	55.4 ± 7.5	58.7 ± 9.5	58.8 ± 11.1	0.331
Sex (male : female)	12 : 13	16 : 11	50 : 18	0.057
Weight (kg)	58.2 ± 15.8	55.1 ± 10.6	63.5 ± 12.7	0.014*
Height (cm)	163.2 ± 9.1	161.5 ± 8.1	159.1 ± 8.9	0.124
Body mass index (kg/m^2^)	21.8 ± 5.1	21.1 ± 3.2	25.0 ± 4.3	<0.001*
Reported NPC stage at diagnosis [27]				
Stage I (*n*, %)	5 (20%)	2 (7.4%)	—	
Stage II (*n*, %)	5 (20%)	7 (25.9%)	—	
Stage III (*n*, %)	11 (44%)	15 (55.6%)	—	
Stage IV (*n*, %)	4 (16%)	3 (11.1%)	—	
Post-NPC duration (year)	12.5 ± 7.1	8.4 ± 9.7	—	0.094
NPC treatment received				
Radiotherapy (*n*, %)	17 (68%)	9 (33.3%)	—	
Radiotherapy and chemotherapy (*n*, %)	7 (28%)	18 (66.6%)	—	
Radiotherapy, chemotherapy, and surgery (*n*, %)	1 (4%)	0 (0%)	—	

The mean ± standard deviation is presented for the continuous variables.

*P < 0.05.

**Table 2:** Comparison of the balance outcomes of the three groups.

	Useful sensory inputs [19]	NPC-TC Qigong group (*n* = 25)	NPC-control group (*n* = 27)	Healthy-control group (*n* = 68)	*P* values		
					NPC-TC Qigong group versus NPC-control group	NPC-TC Qigong group versus healthy-control group	NPC-control group versus healthy-control group
One-leg-stance times							
(1) On ground with eyes open (s)	Somatosensory, visual, and vestibular	12.53 ± 8.40	11.74 ± 11.80	19.80 ± 10.87	1.000	0.013*	0.003*
(2) On stability trainer with eyes open (s)	Visual and vestibular	10.08 ± 10.52	9.00 ± 11.13	16.57 ± 9.71	1.000	0.023*	0.004*
(3) On ground with eyes closed (s)	Somatosensory and vestibular	4.76 ± 6.52	2.81 ± 4.37	8.19 ± 8.83	1.000	0.168	0.007*
(4) On stability Trainer with eyes closed (s)	Vestibular	2.48 ± 2.97	1.48 ± 2.42	5.97 ± 6.64	1.000	0.018*	0.001*
Six-minute walk distance (m)	—	308.00 ± 62.72	285.19 ± 50.34	302.50 ± 63.02	0.528	1.000	0.629

The mean ± standard deviation is presented for all of the variables.

*denotes a significant difference at *P* < 0.05.
